# Metabolic Profiling of Developing Pear Fruits Reveals Dynamic Variation in Primary and Secondary Metabolites, Including Plant Hormones

**DOI:** 10.1371/journal.pone.0131408

**Published:** 2015-07-13

**Authors:** Akira Oikawa, Takao Otsuka, Ryo Nakabayashi, Yusuke Jikumaru, Kanji Isuzugawa, Hideki Murayama, Kazuki Saito, Katsuhiro Shiratake

**Affiliations:** 1 RIKEN Center for Sustainable Resource Science, Yokohama, 230–0045, Japan; 2 Faculty of Agriculture, Yamagata University, Tsuruoka, 997–8555, Japan; 3 Yamagata Integrated Agricultural Research Center, Sagae, 999–7601, Japan; 4 Graduate School of Pharmaceutical Sciences, Chiba University, Chiba, 260–8675, Japan; 5 Graduate School of Bioagricultural Sciences, Nagoya University, Nagoya, 464–8601, Japan; University of Malaga-Consejo Superior de Investigaciones Científicas, SPAIN

## Abstract

Metabolites in the fruits of edible plants include sweet sugars, visually appealing pigments, various products with human nutritional value, and biologically active plant hormones. Although quantities of these metabolites vary during fruit development and ripening because of cell division and enlargement, there are few reports describing the actual dynamics of these changes. Therefore, we applied multiple metabolomic techniques to identify the changes in metabolite levels during the development and ripening of pear fruits (*Pyrus communis* L. ‘La France’). We quantified and classified over 250 metabolites into six groups depending on their specific patterns of variation during development and ripening. Approximately half the total number of metabolites, including histidine and malate, accumulated transiently around the blooming period, during which cells are actively dividing, and then decreased either rapidly or slowly. Furthermore, the amounts of sulfur-containing amino acids also increased in pear fruits around 3–4 months after the blooming period, when fruit cells are enlarging, but virtually disappeared from ripened fruits. Some metabolites, including the plant hormone abscisic acid, accumulated particularly in the receptacle prior to blooming and/or fruit ripening. Our results show several patterns of variation in metabolite levels in developing and ripening pear fruits, and provide fundamental metabolomic data that is useful for understanding pear fruit physiology and enhancing the nutritional traits of new cultivars.

## Introduction

Fruits contain abundant flavor-enhancing and nutritional metabolites that improve human health and longevity. During the development and ripening of fruits, the decomposition of starch produces sugars. At the same time, some types of fruit accumulate colorful metabolites such as anthocyanins and carotenoids or emit fragrant metabolites such as terpenes and phenol esters that attract seed-dispersing birds and animals and help propagate the species. Previous studies on fruits have often focused on metabolites that are directly associated with taste, color, fragrance, and/or nutrition in edible, already ripened fruits [1, 2]. However, fruits also contain many other metabolites that are biologically active, but are not directly associated with fruit quality; moreover, fruits likely contain several unidentified metabolites, particularly species-specific secondary metabolites, similar to those in non-fruiting plants. Furthermore, few studies have examined the changes in metabolite levels in still-developing fruits. A better understanding of metabolic variations during fruit development would potentially improve our understanding of the fundamental biological processes in agriculturally important fruit plants. It would also boost horticultural programs that aim at generating commercially superior fruits with better palatability, nutrient balance, and health benefits.

Plant hormones play critical roles in plant growth and development, fruit ripening and senescence, and physiological responses to biotic and abiotic stresses. Fruit development alone includes a variety of physiological processes, including cell division and enlargement, all of which may rely heavily on plant hormone balance [3]. However, the quantification of plant hormones is more difficult than that of other metabolites because of their much lower concentrations relative to other metabolites. Recent developments in analytical technology have nonetheless enabled the simultaneous detection of most plant hormones [4], thus allowing a broad but thorough analysis. Recently, sugars have been reported as factors that signal fruit ripening [5]. Therefore, metabolomic analyses that includes plant hormones and sugars is necessary to understand fruit physiology during development and ripening.

In this study, we applied a comprehensive analysis of various metabolomic systems to the development and ripening of pear fruits of *Pyrus communis* L. ‘La France’, a rosaceous fruit produced worldwide that has a unique “melting” texture. The cultivar ‘La France’ is the largest pear fruit produced in Japan. Metabolomic analyses included (a) capillary electrophoresis time-of-flight mass spectrometry (CE-TOF MS) for ionic metabolites, including amino acids and organic acids; (b) liquid chromatography quadrupole time-of-flight mass spectrometry (LC-QTOF MS) for neutral metabolites such as polyphenols; (c) liquid chromatography triple quadrupole mass spectrometry (LC-tripleQ MS) for plant hormones; and (d) high-performance liquid chromatography (HPLC) for sugars. These techniques have revealed that there are variations in metabolites, including plant hormones, during fruit development and ripening of pear fruits. Therefore, the final purpose of our study was to identify the physiological roles of metabolites in pear fruits.

## Materials and Methods

### Plant materials

Pear fruits (*Pyrus communis* L. ‘La France’) were harvested from a private orchard in Yamagata Prefecture, Japan (lat. 38°11ʹN and long. 140°28ʹE) in 2010 and 2011 with the permission of the owner. Receptacles were removed at 2 weeks before blooming (2WBB), 1 week before blooming (1WBB), the time of blooming (B), and 2 weeks after blooming (2WAB). Furthermore, peeled and deseeded fruits were collected at 1 month after blooming (1MAB), 2 months after blooming (2MAB), 3 months after blooming (3MAB), 4 months after blooming (4MAB), and the time of harvesting (H; 5 months after blooming) and 1 month after harvesting (1MAH; ripened fruits) and used for subsequent metabolomic analyses. These samples were transferred from the orchard to the laboratory as quickly as possible and were then frozen in liquid nitrogen. The frozen samples were weighed and then crushed using a homogenizer. The crushed pear fruits were placed in conical tubes and stored at –80°C until extraction. At least three biological replicates were prepared for each sample.

### Sample extraction

Crushed and frozen samples (10 g) were resuspended in 50 mL of MeOH, including internal standards for standardization of peak areas, and centrifuged (16,000 × *g*, 3 min, 4°C). Supernatants were dispensed for each analysis as follows: 0.5 mL for ionic metabolites, 1 mL for neutral metabolites, 1 mL for sugars, and 47.5 mL for plant hormones. Because of the small amounts of pear fruit samples obtained in the early periods (2WBB, 1WBB, and B), the initial amounts of these samples were 1 g, extracted in 20 mL of MeOH, and were separately used for each analysis as described above. The residues were subsequently used for starch analysis.

### Metabolite profiling using CE-TOF MS

For the analysis of ionic metabolites, hydrophobic and high molecular weight compounds were removed by the preparative processes of liquid–liquid separation using chloroform and water, and ultrafiltration using a 5 kDa cutoff filter, respectively, prior to the metabolomic analyses [6]. Comprehensive analysis of ionic metabolites using CE-TOF MS was performed as previously reported [7].

### Untargeted metabolome analysis by LC-QTOF-MS

Extracts (1 mL) containing the internal positive and negative standards 2.5 μM lidocaine and 10-camphorsulfonic acid, respectively, were analyzed using LC-QTOF-MS (LC, Waters Acquity UPLC system; MS, Waters Xevo G2 Q-Tof). Analytical conditions were as follows: LC column, Acquity bridged ethyl hybrid (BEH) C18 (1.7 μm, 2.1 mm × 100 mm, Waters); solvent system, solvent A (water containing 0.1% formic acid) and solvent B (acetonitrile containing 0.1% formic acid); gradient program, 99.5%A/0.5%B at 0 min, 99.5%A/0.5%B at 0.1 min, 20%A/80%B at 10 min, 0.5%A/99.5%B at 10.1 min, 0.5%A/99.5%B at 12.0 min, 99.5%A/0.5%B at 12.1 min and 99.5%A/0.5%B at 15.0 min; flow rate, 0.3 mL/min; column temperature, 40°C; MS detection: capillary voltage, +3.0 keV, cone voltage, 25.0 V, source temperature, 120°C, desolvation temperature, 450°C, cone gas flow, 50 L/h; desolvation gas flow, 800 L/h; collision energy, 6 V; mass range, m/z 100‒1500; scan duration, 0.1 s; interscan delay, 0.014 s; mode, centroid; polarity, positive; Lockspray (leucine enkephalin): scan duration, 1.0 s; interscan delay, 0.1 s. The data matrix was aligned with MassLynx version 4.1 (Waters). After alignment, deisotoping, and cutoff of low-intensity peaks (fewer than 500 counts), intensity values of the remaining peaks were divided by those of lidocaine ([M+H]+, *m/z* 235.1804) and 10-camphorsulfonic acid ([M-H]-, *m/z* 231.06910) for normalization. MS/MS data were acquired in ramp mode under the following analytical conditions: (1) MS: mass range, *m/z* 50–1500; scan duration, 0.1 s; interscan delay, 0.014 s; and (2) MS/MS: mass range, *m/z* 50–1500; scan duration, 0.02 s; interscan delay, 0.014 s; data acquisition, centroid mode; collision energy, ramped from 10 to 50 V. In this mode, MS/MS spectra of the top 10 ions (>1000 counts) in an MS scan were automatically obtained. When the ion intensity was less than 1000, MS/MS data acquisition was not performed. The secondary metabolites were chemically assigned by deciphering MS/MS spectra [8].

### Sugar and starch analysis

One milliliter of the MeOH extract obtained for sugar analysis was evaporated to dryness and dissolved in 0.75 mL of water. The same volume of 1% (w/v) mannitol (as an internal standard) solution was added to the sample solution, and this mixture was used for subsequent sugar analysis. Sugars (glucose, fructose, sucrose, and sorbitol) were quantified by HPLC according to the conditions described below. The analytical conditions of HPLC were as follows: column, Shim-pack SCR101-C column, 70°C; solvent, distilled water; flow rate, 1 mL/min, isocratic; detection, RI detector (HITACHI L-7490). For starch analysis, the residues of sample extracts were used. An alcohol-insoluble residue was prepared using the method described by Murayama *et al*. [9]. For starch quantification, the dried alcohol-insoluble residue was first suspended in distilled water and boiled for 30 min. After cooling, the gelatinized starch was digested with amyloglucosidase (from *Aspergillus niger*; Roche Applied Science) in 50 mM sodium acetate buffer (pH 4.5). The released glucose was measured using the glucose oxidase–peroxidase method of Barham and Trinder [10].

### Quantification of plant hormones using LC-tripleQ MS

Fifteen plant hormones were detected and quantified using stable isotopes of each hormone and LC-tripleQ MS analyses were performed as previously reported [6]. Briefly, methanol extracts were evaporated and resuspended in 5 volumes of 80% MeCNaq. containing 1% AcOH with internal standards and deuterated plant hormones, for quantification, and then extracted by mixing occasionally on ice for 1 h. After centrifugation, the pellet was resuspended and extracted with the same volume of the solvent described above, and supernatants were mixed. The MeCN was evaporated and the residual solution was purified using solid-phase extraction columns. Brassinosteroids required further purification by HPLC.

### Metabolomic data analysis and statistics

Data describing the quantified or relative amounts of metabolites were integrated into one sheet. These data were standardized by subtraction of the averages from each amount and division of the resulting values by the standard deviations. The standardized data were subjected to principal component analysis (PCA) and hierarchical clustering analysis (HCA) using DrDMass (http://kanaya.naist.jp/DrDMASS/) [11] and PermutMatrix (http://www.lirmm.fr/~caraux/PermutMatrix/) [12], respectively. In HCA, Euclidean distance and Ward’s minimum variance were used as a dissimilarity and a linkage rule, respectively.

### Analysis of protein-derived amino acids

Crushed and frozen samples (500 mg) were resuspended in 1 mL of 50 mM phosphate buffer (pH 7.0), maintained on ice for 30 min, and then centrifuged (12,000 × *g*, 30 min, 4°C). The supernatant (400 μL) was mixed with 1.2 mL cold acetone and placed in a freezer at –30°C for 120 min. After centrifugation of the mixture (12,000 × *g*, 5 min, 4°C), the supernatant was carefully discarded and the pellet was suspended in 600 μL of water.

Performic acid reagent was prepared by mixing 30% (w/w) H_2_O_2_ and 98% (w/w) formic acid at a ratio of 1 to 9. The solution was mixed for 1 h at room temperature [13, 14]. The reagent (12.5 mL) was added to the sample solution (600 μL) and refrigerated at 4°C for 16 h. Most of the reagent was then removed under reduced pressure using a rotary evaporator (N-1000; EYELA, Tokyo, Japan). After evaporation, the sample was resuspended in 15 mL of 6 N HCl and then transferred to a screw-capped tube. The tube was placed in an oil bath (HOA-50A; ASONE, Tokyo, Japan) at 130°C for 20 h. The reagent was again removed under reduced pressure on a rotary evaporator and dissolved in 1 mL of MeOH. A total of 500 μL of this MeOH solution was used for CE-TOF MS analysis as described above. Total protein was quantified by the Bradford method [15].

## Results

### Pear fruit development

**Fig 1:**
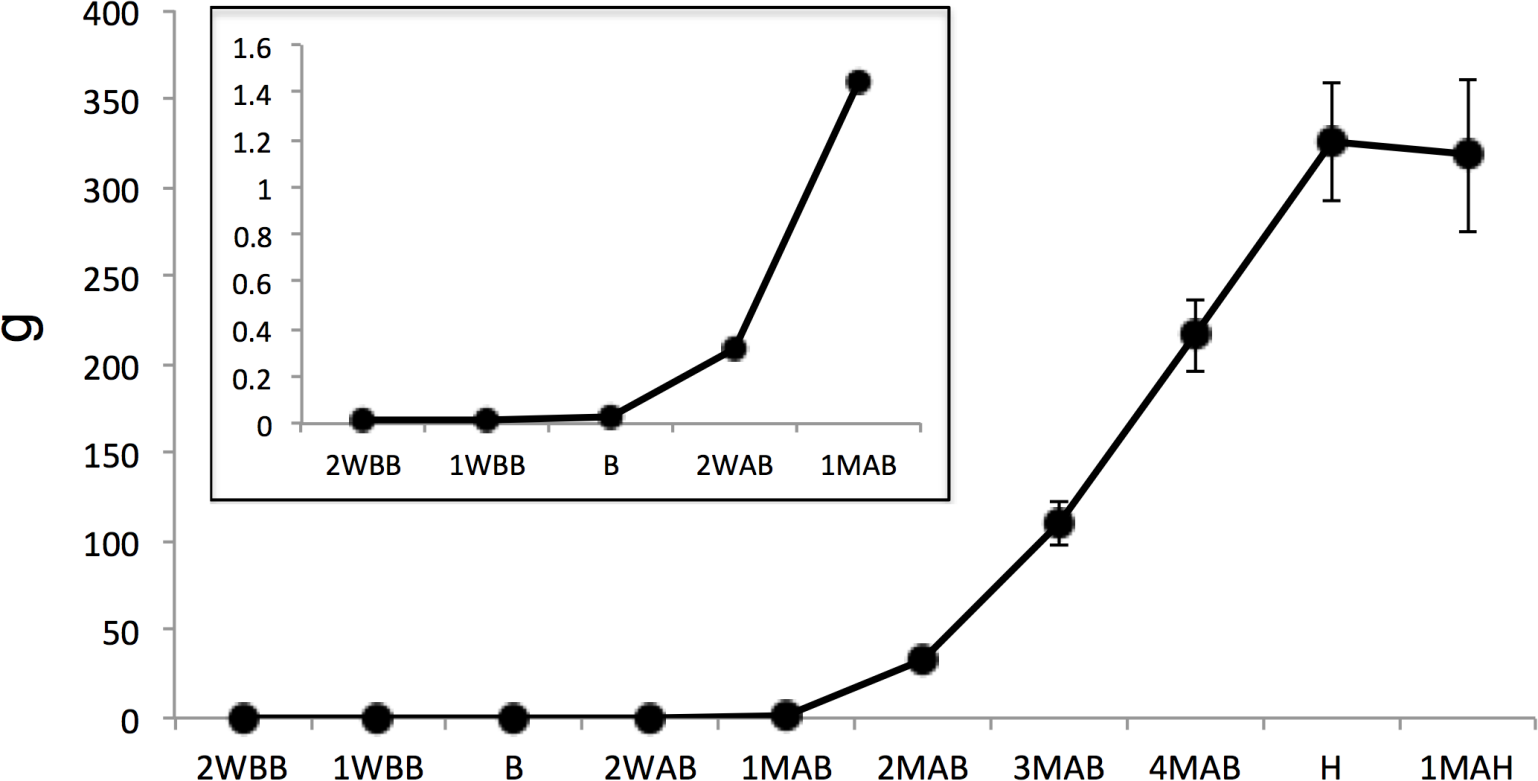
Fresh weight of pear fruits during development and ripening. Sampling times were at 2 weeks before blooming (2WBB), 1 week before blooming (1WBB), the time of blooming (B), 2 weeks after blooming (2WAB), 1 month after blooming (1MAB), 2 months after blooming (2MAB), 3 months after blooming (3MAB), 4 months after blooming (4MAB), the time of harvesting (H), and 1 month after harvesting (1MAH). The values from 2WBB to 1MAB are shown in the inset. Error bars indicate standard deviations of three replicates.

Fruit weights determined at each sampling point were used to characterize the development of pear fruits over time (Fig 1). Samples taken at 2WBB, 1WBB, and the time of B showed only slow development of the receptacle, which is the part that grows into the actual fruit (0.012 ± 0.0016 g, 0.021 ± 0.0010 g, and 0.024 ± 0.0011 g, respectively, presented as mean ± standard deviation). At 2WAB, the fruit weight had rapidly increased to 0.32 ± 0.014 g. The fruit developed at a similar rate until the time of H, leading to an approximately 13,500-fold increase in the fruit weight compared to the B sample. In the ripening sample taken at 1MAH, the average fruit weight decreased slightly, although not significantly.

### Metabolomic analyses of pear fruits

For time-course metabolomic analyses, pear fruits were extracted with methanol and further prepared depending on the analytic technique used. Using CE-TOF MS, 218 ionic metabolites, including amino acids, organic acids, nucleosides, nucleotides, sugar phosphates, and polyamines, were detected in pear fruits. LC-TOF MS detected 18 neutral metabolites, including flavonoids. Fifteen plant hormones were quantified using LC-tripleQ MS. The three primary sugars (glucose, fructose, and sucrose), a sugar alcohol (sorbitol), and starch were quantified using HPLC. S1 Fig lists the full set of analytes. In addition, all metabolite data can be browsed and downloaded from our database, the “Fruits Omics Database” (http://www.tr.yamagata-u.ac.jp/~oikawa/oikawa/GLPDB%202/GLPDB_E/index.html).

### Annual changes in the pear fruit metabolome

**Fig 2:**
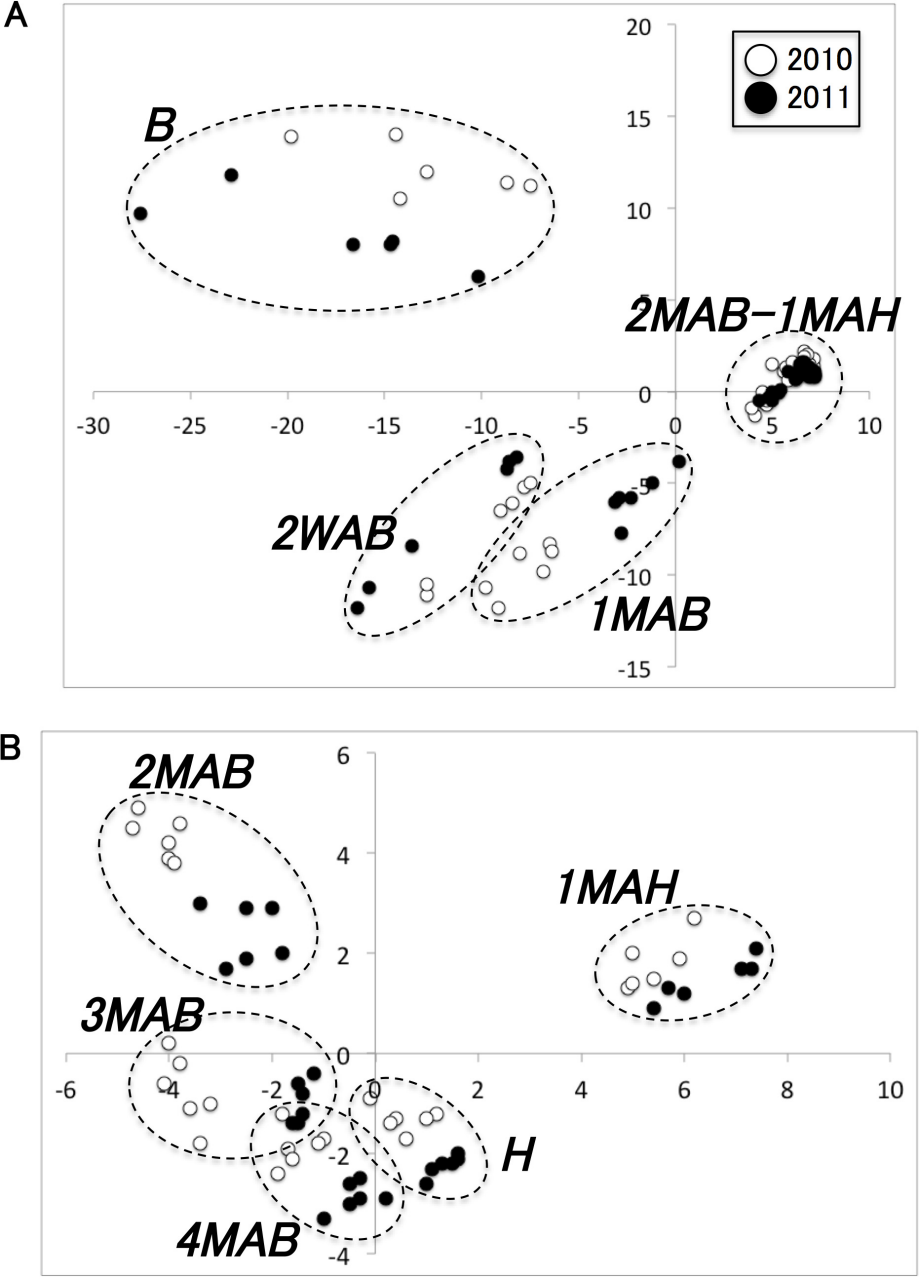
Principal component analysis (PCA) of metabolome data. (a) Data obtained from pear fruits after blooming in 2010 (open circles) and 2011 (closed circles). The horizontal and vertical axes represent principal component 1 (PC1, contribution ratio 45.7%) and 2 (PC2, 18.1%), respectively. (b) Data for pear fruits from 2MAB onward during the same 2 years. The horizontal and vertical axes represent PC1 (33.9%) and PC2 (14.9%), respectively.

The orchard-grown fruit used in this study was exposed to annual and seasonal changes in weather. We performed metabolomic analyses of pear fruits for 2 years, 2010 and 2011, to investigate annual/seasonal differences in pear fruit metabolites. PCA of the metabolomic data obtained from samples from 2010 and 2011 was used to visualize these changes (Fig 2). Because pre-B samples were not obtained in the 2010 experiments, only B and post-B data points were compared. When visualizing the PCA, multivariate metabolome data are represented in two dimensions. Each circle in the figure represents one sample, and the distance between two circles indicates the similarity of metabolite varieties and amounts in the respective samples. Therefore, closer circles represent greater similarity. As shown in Fig 2A, PCA showed large differences between the samples from the time of B and the samples from either at 2WAB or 1MAB. In this PCA, samples obtained from 2MAB to 1MAH were not separated. Therefore, we again performed PCA using data of samples from 2MAB to 1MAH (Fig 2B). At most sampling points, there were differences between the 2010 and 2011 data, representing annual differences. However, these annual differences were smaller than the seasonal differences among sampling points. The first principal component in Fig 2B shows the changes in metabolite levels during ripening (1MAH). Metabolites with high positive loading scores on this axis, including glucose, ABA, and Pro, were inferred to be factors associated with ripening.

### Metabolite classification based on patterns of variation during development and ripening

**Fig 3:**
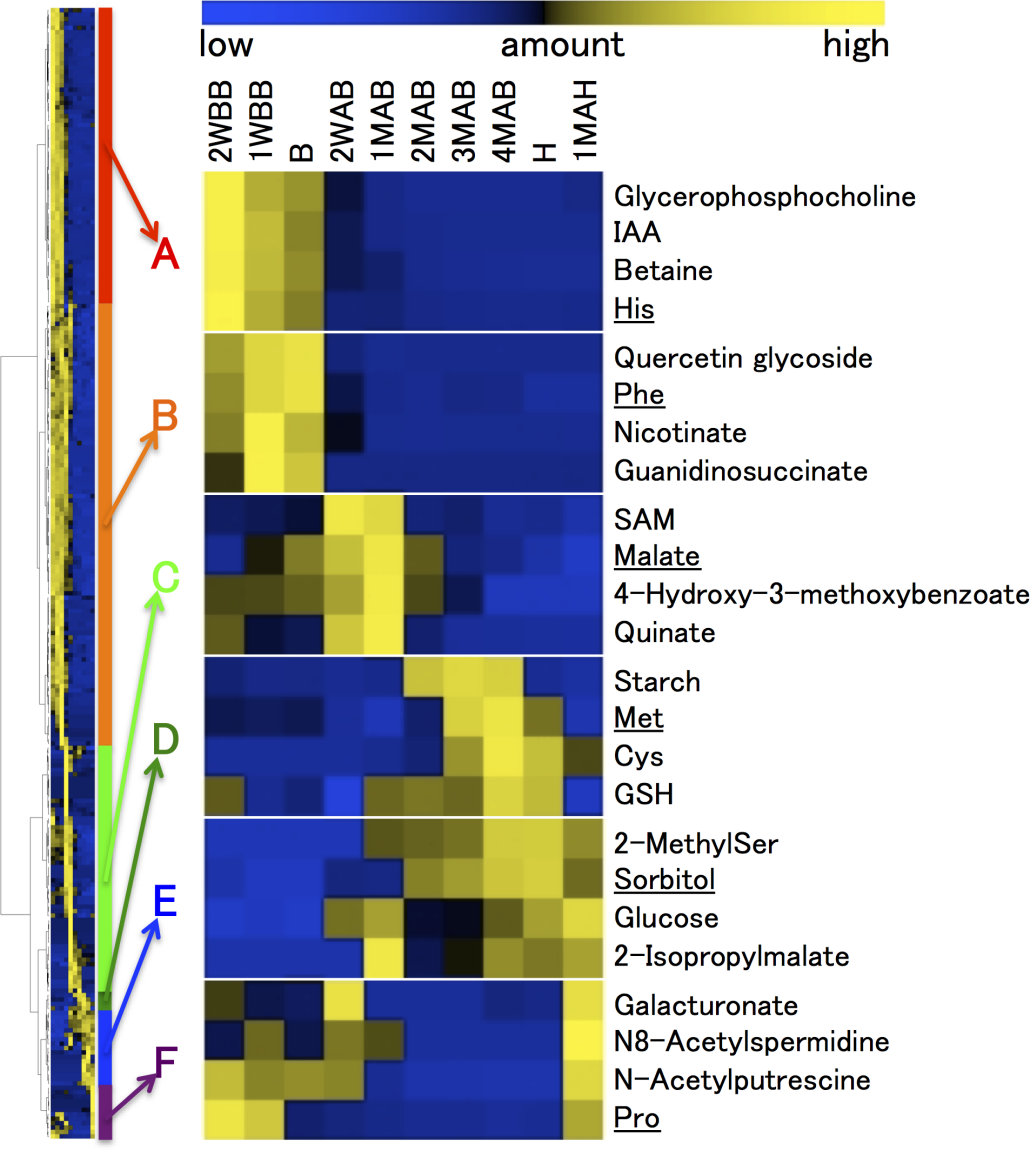
Hierarchical cluster analysis (HCA) of metabolome data of pear fruits from 2WBB to 1MAH in 2011. Yellow and blue colors represent higher and lower amounts of metabolites, respectively. Details of HCA are shown in S1 Fig Depending on the variation patterns of metabolite amounts, detected metabolites were classified into 6 groups, A to F. The enlarged figures show HCA results of four representative metabolites in each group. The detailed changes in the amounts of the underlined metabolites in the enlarged graphs are shown in Fig 4.

**Fig 4:**
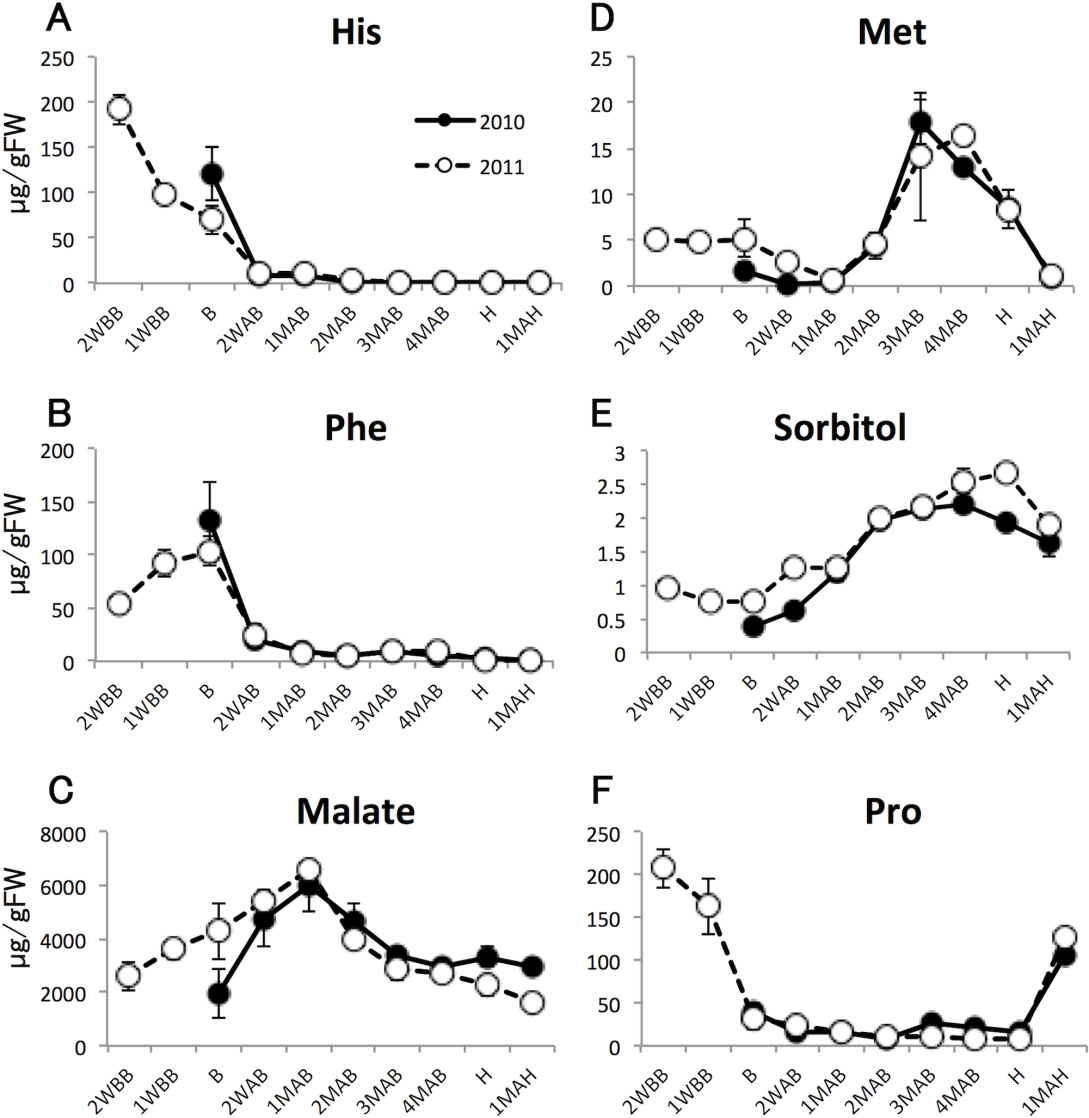
Amounts of representative metabolites of each group. Panels A to F correspond to groups A to F as presented in Fig 3. Closed and open circles indicate 2010 and 2011 samples. Error bars indicate standard deviations of three replicates.

**Fig 5:**
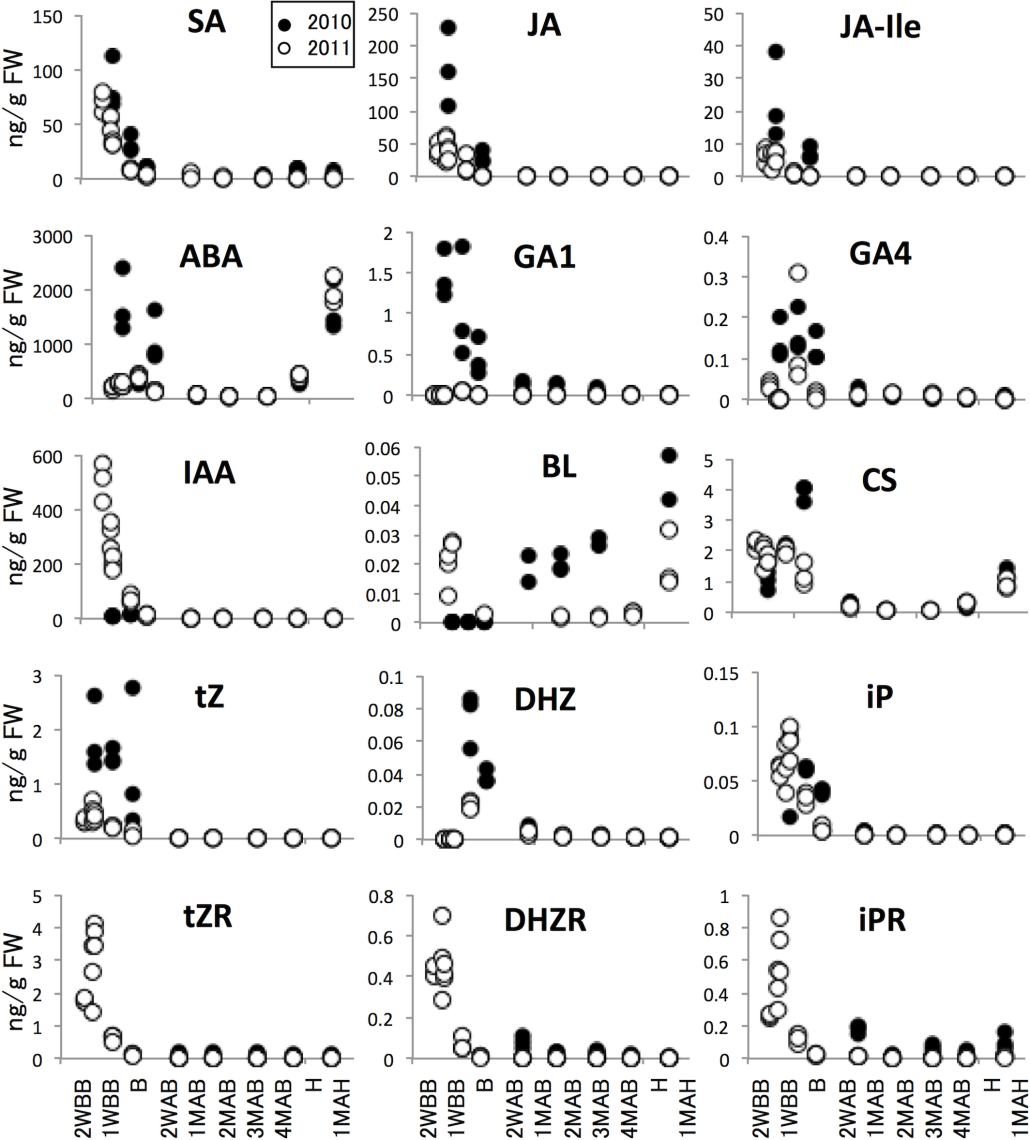
Time-course changes in amounts of plant hormones in developing and ripening pear fruits. Closed and open circles indicate 2010 and 2011 samples. Abbreviations: SA, salicylic acid; JA, jasmonic acid; JA-Ile, jasmonic acid Ile conjugate; ABA, abscisic acid; GA1, gibberellin A1; GA4, gibberellin A4; IAA, indole acetic acid; BL, brassinolide; CS, castasterone; tZ, *trans*-zeatin; DHZ, dihydrozeatin; iP, isopentenyladenine; tZR, *trans*-zeatin riboside; DHZR, dihydrozeatin riboside; iPR, isopentenyladenine riboside.

**Fig 6:**
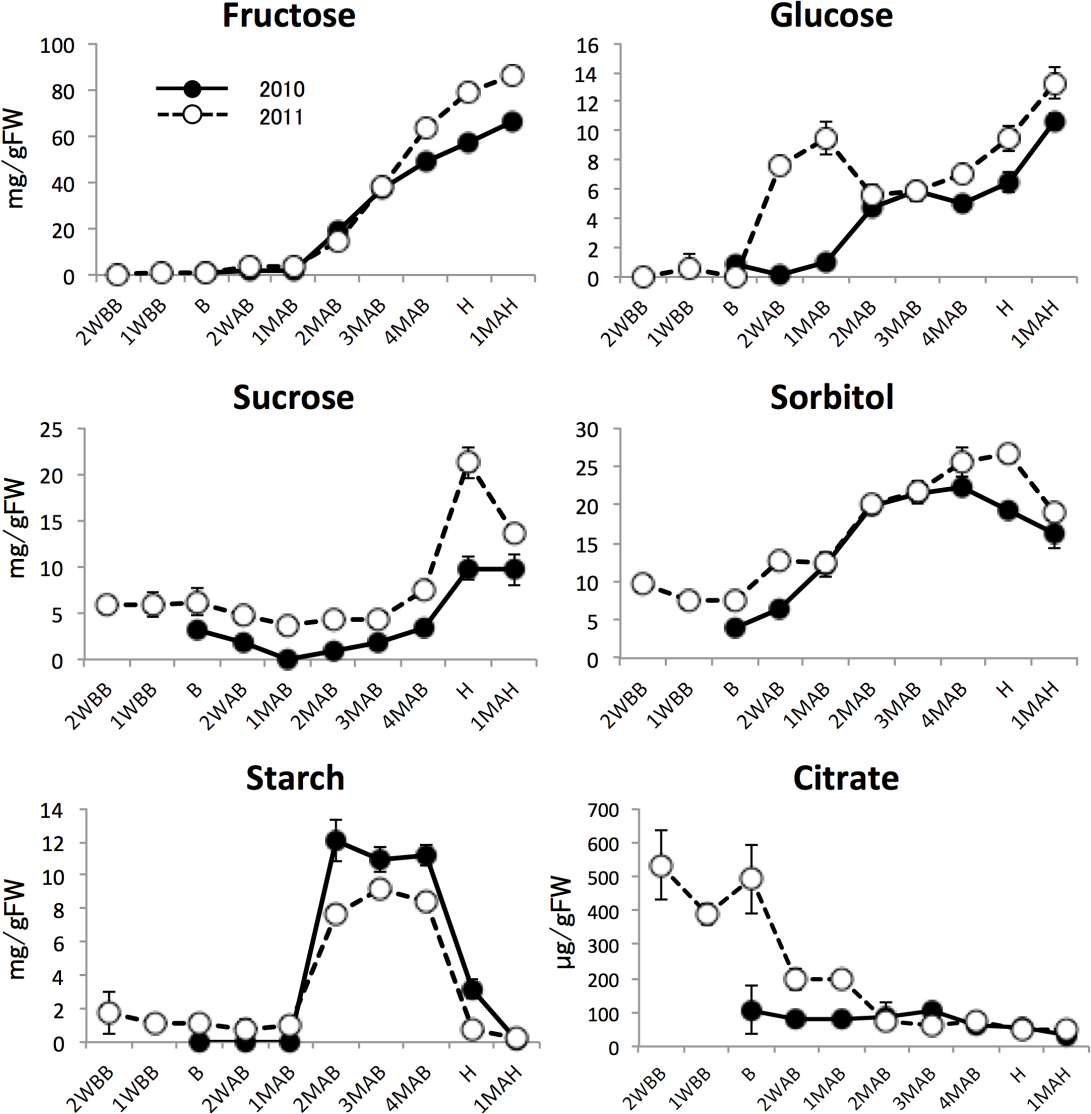
Changes in amounts of sugars, starch, and citrate. Closed and open circles indicate 2010 and 2011 samples. Error bars indicate standard deviations of three replicates.

HCA of the 2011 metabolomic data was used to characterize seasonal changes (Fig 3 and S1 Fig). We then classified all analytes into six groups (A to F) according to patterns of time-dependent changes in metabolite levels (Fig 3). Detailed descriptions of each group and some of their metabolites follow and include data from 2010 where available. After analyzing the seasonal changes and classifying the studied metabolites, we chose representative metabolites from each group (the underlined metabolites in Fig 3) to document the 2010–2011 annual differences at and following the time of B, as shown in Fig 4 (panel labels A–F correspond to group labels A–F). The annual differences were substantially smaller than the seasonal differences. Moreover, plant hormone levels changed in developing pear fruits harvested in 2010 and 2011 (Fig 5). The metabolic dynamics of four sugars, starch, and citrate detected in this study are also shown in Fig 6.

#### Groups A and B

The levels of metabolites that were classified into group A showed marked decreases from 2WBB and no increase after the time of B. This group included some free amino acids such as His, Ser, *O*-acetyl Ser, Arg, and Gln and plant hormones such as indole-3-acetic acid (IAA), jasmonic acid (JA), and JA-isoleucine (JA-Ile). In contrast, the levels of metabolites in group B peaked at the time of B, and decreased markedly thereafter. This group could be differentiated from group A only in the 2011 data because the main difference between the two was in the levels of pre-B metabolites. Group B contained free hydrophobic amino acids such as Phe and Val, conjugated phenylpropanoids, including chlorogenate and procyanidin, and many sugar phosphates.

#### Group C

These analytes were characterized by rapid increases in the levels of metabolites after B, followed by moderate to rapid decreases. The periods when these metabolite levels increased (2WAB and 1MAB) corresponded roughly to the periods of fruit cell division and seed formation. Simple phenylpropanoids such as quinate and (epi)catechin; polyamines, including spermine and putrescine; and a principal organic acid in pear fruits, malate, were classified in this group.

#### Group D

**Fig 7:**
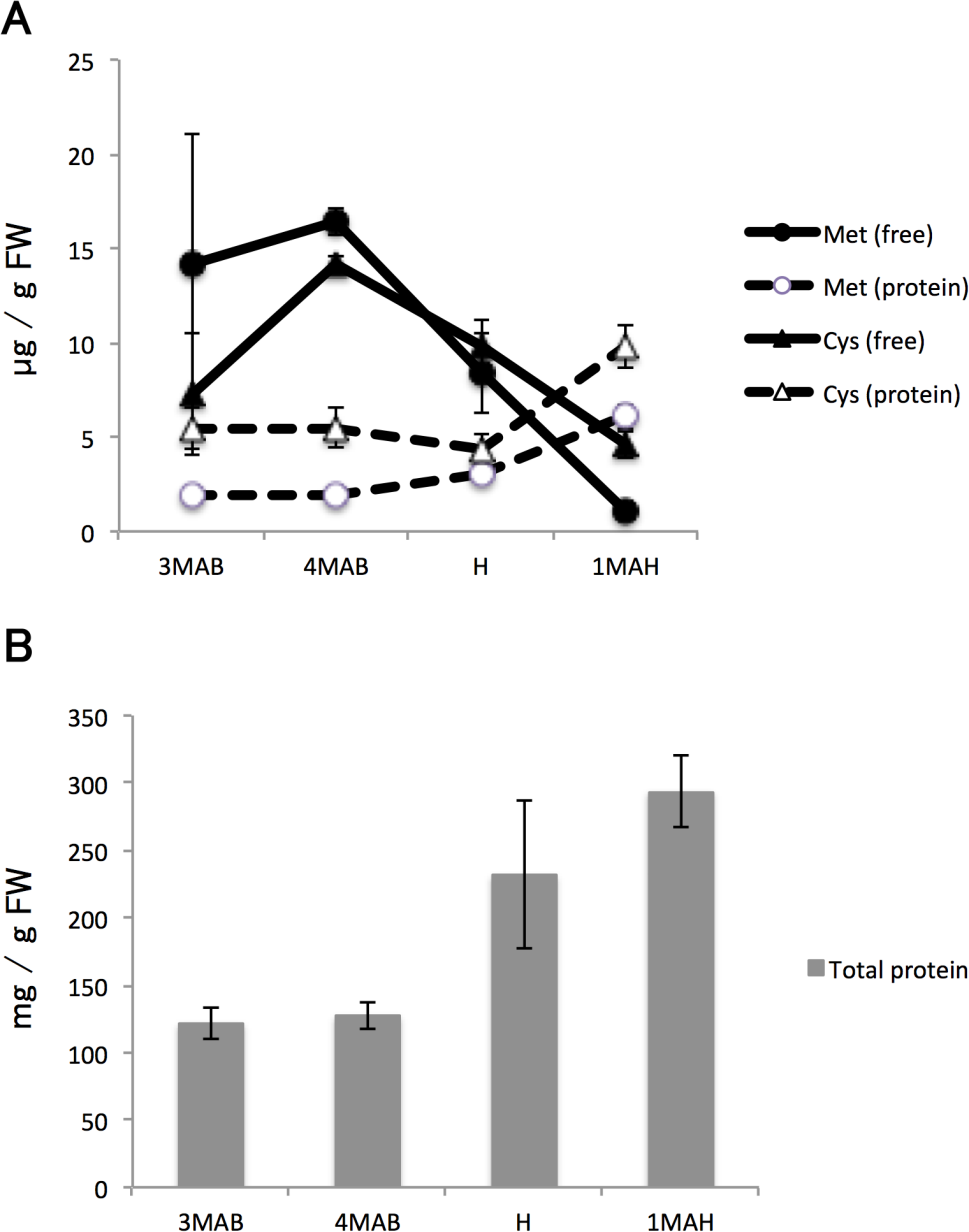
Protein and sulfur-containing amino acid levels in pear fruits. (A) Amounts of free and protein-derived Met and Cys in pear fruits at 3MAB, 4MAB, H, and 1MAH. Closed and open circles and triangles represent free and protein-derived Met and Cys, respectively. (B) Total protein amounts in pear fruits at the same sampling times as (A). Error bars indicate standard deviations of three replicates.

In general, the levels of metabolites in group D peaked transiently around 3–4MAB and declined rapidly thereafter; however, unexpected variations were also noted in this group (note the high standard error at 3MAB in Fig 4D). The accumulated metabolites were undetectable in the ripening fruits at 1MAH. This group included sulfur-containing metabolites such as Met, Cys, and GSH as well as starch. To test the hypothesis that these amino acids are used for protein synthesis in ripening fruits, we quantified the sulfur-containing amino acids originating in proteins (Met and Cys) as well as the total protein contents in the 2011 samples both after B and around the time of H. Changes in the levels of free and protein-derived Met and Cys are shown in Fig 7 in addition to the total protein content in the pear fruit at 3 and 4 months after B, at the time of H, and 1 month after H. The levels of free Met and Cys decreased, whereas protein-derived levels increased during ripening, suggesting that free Met and Cys are used to synthesize protein during this period. At the same time, total protein also accumulated during ripening. However, our data did not permit determination of the amount of free Met and Cys that contribute to protein accumulation in ripened fruits.

#### Group E

The levels of group E metabolites changed synchronously with fruit weight (see Fig 1). Group E included sugars derived from starch degradation, such as glucose. Sorbitol, a translocated sugar alcohol found in rosaceous fruits, was also classified in this group.

#### Group F

The metabolites in this group were separated into two subgroup; those that increased particularly in ripening fruits and those that accumulated both before B and during ripening. ACC and ABA represent the former subgroup. Proline is characteristic of the latter subgroup.

### Changes in concentrations of plant hormones in developing and ripening pear fruits

Because plant hormones regulate morphogenesis, including seed formation and ripening, we analyzed variation in the concentrations of various plant hormones that were detectable in pear fruits over time. The equipment detected 15 plant hormones, including one auxin, six cytokinins, two gibberellins, one abscisic acid, one salicylic acid, two jasmonic acids, and two brassinosteroids. Changes in the concentrations of these plant hormones during development and ripening of the pear fruits were quantified (Fig 5). It is more difficult to analyze plant hormones quantitatively than other metabolites because of the very small quantities that are naturally present and the relative ease with which environmental and/or artificial stimuli induce variations in hormone levels. These features are represented in the data as wide variations in concentrations that were detected within the same time group. However, with the exception of these deviations, certain dynamic characteristics were evident during fruit development and ripening (Fig 5). Several plant hormones, including IAA, accumulated before B and decreased drastically after B. Majority of plant hormones showed very low levels until H, and only ABA and brassinosteroid levels increased after H. In addition to ABA, certain other plant hormones showed unexpected and interesting accumulation patterns in the pear fruit (Fig 5). Although few data exist to explain the purpose of these hormone accumulations, particularly the very high accumulation of IAA before B and the increase in brassinosteroid levels after H, our data suggest novel roles for plant hormones in fruit development and ripening.

## Discussion

Conventional metabolite analyses of fruits have focused on several metabolites associated with bioactivity, such as plant hormones [16, 17], or fruit color and/or taste, such as anthocyanins or sugars [18, 19]. Recently, metabolomic data have been reported for tomato [20, 21], peach [22], strawberry [23, 24], and grape [25, 26]. These comprehensive studies revealed that there are dynamic variations in the levels of many metabolites, including amino acids, sugars, organic acids, and phenylpropanoids, during fruit development. In addition to quantifying these metabolites in pear fruits, we quantified the levels of various plant hormones, including trace levels of brassinosteroids. This broad-spectrum target analysis was accomplished through a combination of multiple analytical techniques, CE-TOF MS, LC-TOF MS, LC-tripleQ MS, and HPLC, to achieve a survey of pear fruit metabolites that was as comprehensive as possible. More than 250 metabolites were detected in samples from developing and ripening pear fruits, which was a number equal to the maximum number of metabolites that was studied in previous fruit metabolome analyses. Addition of further analytical instruments such as a GC-MS, which is suitable for the comprehensive analysis of volatiles, would permit compilation of an even more comprehensive metabolome dataset.

The asynchronous variation of amino acid levels in pear fruits during fruit development and ripening suggested that amino acids play various roles, depending on their biological properties. For example, Phe is an initial compound in the process of phenylpropanoid biosynthesis (including flavonoids) and in our study it was observed to accumulate until B (group B). Following the sharp decrease in Phe after B, the level of simple phenylpropanoids such as (epi)catechin increased (group C), suggesting that some of these phenylpropanoids were synthesized from the previously accumulated Phe. At the same time, quinate, a precursor of chlorogenate with caffeate, also accumulated. Interestingly, conjugated phenylpropanoids such as procyanidin and chlorogenate were also classified in group B. These conjugated phenylpropanoids appeared to be biosynthesized and accumulated in pear fruits prior to B, and were then degraded into quinate and simple phenylpropanoids following B, thereby contributing to their accumulation. The roles of these simple phenylpropanoids in fruit after B remain to be determined.

Changes in the amounts of organic acids, which are also essential to fruit taste, in developing and ripening pear fruits were different from those reported in other fruits. Malate, an abundant organic acid in pear fruits, showed a gradual increase until 1MAB, after which it decreased (Fig 4C). Pear fruits contain considerable amounts of citrate, although in developing fruits the level of citrate was approximately one tenth that of malate. In this study, citrate amounts were found to decrease toward harvest, although the amounts in pear fruits harvested in 2011 were higher than those in 2010 (Fig 6). In tomato, the amount of malate during fruit development changed in a manner similar to that in pear fruits. However, citrate increased markedly during the late developmental stages [21]. Furthermore, in strawberry, both malate and citrate showed simple increases with advancing fruit maturity [23]. The level of malate in developing peach fruits did not change significantly, whereas citrate showed complex variations in both developing and ripening fruits. The amount of citrate increased after B until approximately 2 weeks before H, then decreased until 3 days after H, and then increased 5 days after H [22]. Such differences between fruits suggest that dynamic changes in the levels of organic acids can be used to characterize each fruit.

Levels of sulfur-containing amino acids, Cys and Met, accumulated transiently at 3–4MAB (Fig 3, group D). This phenomenon has not been reported in previous fruit metabolome studies. In tomato, strawberry, peach, and grape, the levels of Met did not change significantly [21–23, 25]. Indeed, in these studies, the levels of Cys during development were either unchanged or undetectable. Results from the protein-derived amino acid and total protein analyses suggested that the accumulated free Cys and Met were used for protein synthesis.

**Fig 8:**
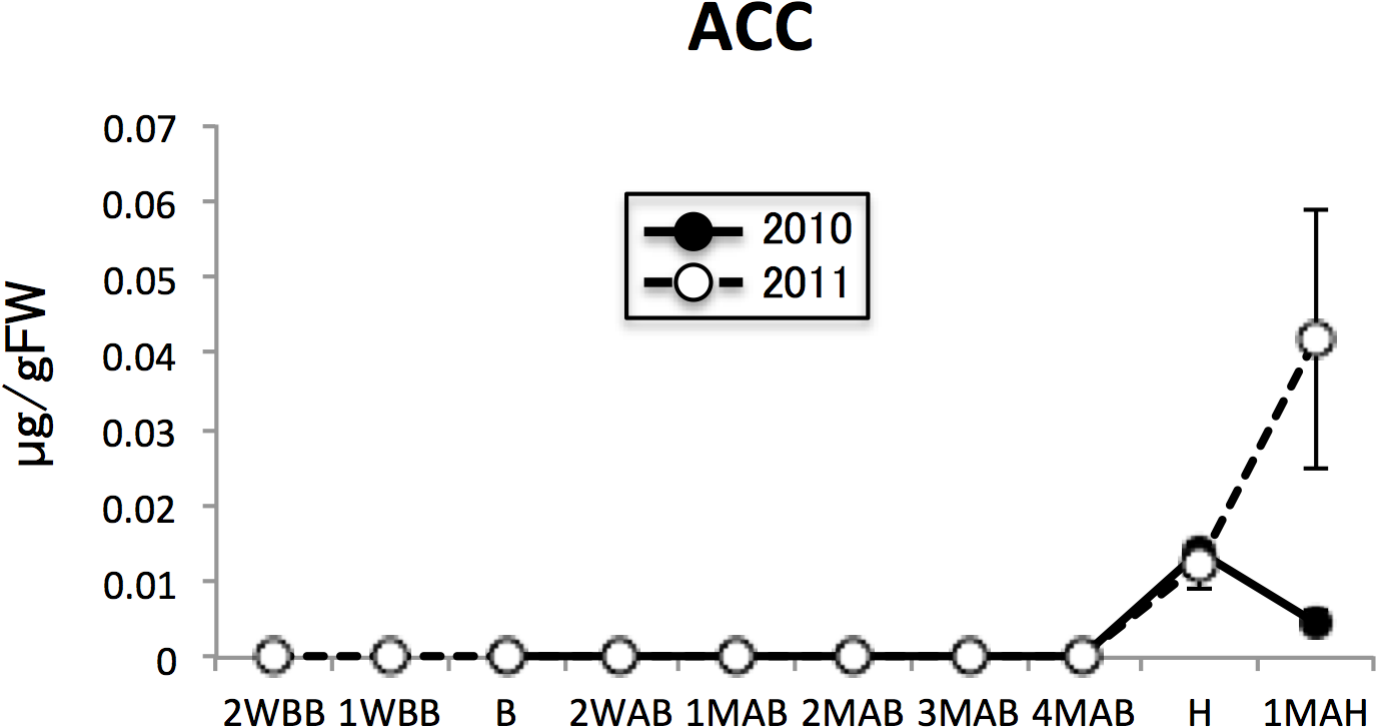
ACC levels in pear fruit during development and ripening. Closed and open circles indicate 2010 and 2011 samples. Error bars indicate the standard deviations of three replicates.

Met is a precursor in the synthesis of ethylene, a gaseous signaling molecule associated with ripening, particularly in climacteric fruits. The increase persisted into 1MAH in the 2011 samples, albeit with high variability, but decreased sharply in the 1MAH samples of the 2010 fruit. Therefore, although some of the Met used for ethylene biosynthesis is recycled by the methionine salvage pathway [27], the decrease in Met in the pear fruits toward H may be associated with the biosynthesis of ethylene, either instead of or in addition to protein synthesis. In fact, in our study, the levels of the ethylene precursor 1-aminocyclopropane-1-carboxylic acid (ACC) markedly increased in pear fruits after and at the time of H (Fig 8). Some of the accumulated Met is assumed to be used for ethylene biosynthesis via ACC. However, met accumulation was not observed before H in tomato or peach, which are also climacteric fruits [21, 22]. The possibility also exists that the accumulation of Met is a species-specific phenomenon. However, we have no metabolomic data for pear cultivars other than the ‘La France’ used in this study.

The Cys-containing tripeptide glutathione (GSH) showed a variation pattern similar to that of Cys (Fig 3). In plants, GSH plays various roles and is involved in redox homeostasis, detoxification of toxic xenobiotics, regulation of the cell cycle, and storage of organic sulfur [28, 29]. The temporal accumulation of GSH may reflect these roles in pear fruits. In addition to studies in multiple pear varieties, future metabolomic analyses of other developing fruits may reveal the biological significance of the accumulation of sulfur-containing amino acids in fruits.

Sugars are important determinants of fruit quality. In our study, the levels of fructose and glucose were found to increase continuously after 2MAB (Fig 6). Given that starch in pear fruits transiently accumulates from 2MAB to 4MAB, the increases observed in fructose and glucose until 4MAB are probably because of both translocation from leaves and the degradation of starch. In contrast, increases in these sugars and in sucrose at the time of H and 1MAH (Fig 6) could be exclusively because of starch degradation. The concentration of sorbitol increased continuously from 2MAB to the time of H and then decreased in ripening fruits (Fig 6). Sorbitol dynamics in pear fruits could be because of an increase resulting from translocation in the developing stage, with a decrease caused by the removal of fruits from a branch during the time of H. The varying patterns of sugars during fruit development identified in this study are similar to those observed in other fruits [21–23].

In climacteric fruits, ethylene is a key plant hormone associated with fruit ripening. However, some reports have implicated ABA in fruit development and ripening in non-climacteric fruits such as strawberry [3, 30]. Ripening fruits also accumulate sugars such as sucrose, leading to increased osmotic pressure and loosening of the cell walls, a phenomenon exacerbated by water loss in fruits after H. These stresses induce ABA or Pro accumulation in plants [31–33]. Therefore, the accumulation of ABA and Pro in pear fruits after H may be associated with osmotic stresses and/or fruit ripening. A time-course analysis of plant hormones in non-ripening climacteric fruits such as tomato, and non-climacteric fruits such as peach, may potentially reveal the roles of ABA in fruit development and ripening.

We also observed specific increases in brassinosteroid levels during pear fruit ripening. In previous reports, brassinosteroids have been suggested to be involved in the ripening of tomato pericarp and grape berry [34, 35]. Our data may also show novel physiological roles of brassinosteroids in fruit ripening.

Fruits are a very palatable and important as well as relatively low-cost source of nutrition. Horticultural scientists accordingly use genetic approaches to improve fruit qualities such as yield, color, taste, and disease resistance. Quantitative trait locus (QTL) analysis is a powerful tool that is used to identify markers for breeding purposes when further knowledge of the underlying genes is lacking [36]. Metabolomic data are also used for QTL analysis using a variant called mQTL [37–40]. Although the pear genome was sequenced very recently [41], detailed functional analysis of most genes is still lacking. In the near future, this metabolomic data may help to advance the mQTL analysis of pears, thus speeding up the development or identification of new ways to develop improved pear cultivars.

We used the most popular pear cultivar in Japan, "La France." Other cultivars that show different phenotypes such as skin color and taste may also differ in metabolic dynamics during maturation and ripening, given that the skin color and fruit taste are directly connected to amounts of metabolites such as carotenoids or sugars. Therefore, other cultivars with color and taste differing from those of ‘La France’ probably have different varieties and dynamics of metabolites. In addition to annual changes, future studies using other pear cultivars will clarify more comprehensively the dynamics of metabolites in developing and ripening pear fruits.

## Conclusion

We described the metabolomic changes occurring in developing and ripening pear fruits. The variations of several metabolites, including sugars, organic acids, some amino acids such as Phe and Pro, and ABA were similar to those in previous reports. However, unexpected changes in the amounts of sulfur-containing amino acids and brassinosteroids may imply novel physiological roles of their metabolites in the development and ripening of pear fruits. Compared to other rosaceous fruits that display dynamic changes in metabolites during development and ripening, some metabolites in pear fruits showed similar accumulation patterns. However, specific dynamics of metabolites also appeared in pear fruits. Future studies using other fruits will reveal the dynamic changes of metabolites in developing and ripening fruits.

## Supporting Information

S1 Fig Hierarchical cluster analysis (HCA) of metabolome data of pear fruits from 2WBB to 1MAH in 2011. Yellow and blue colors represent higher and lower amounts of metabolites, respectively.
(JPG)

## References

[1] Klee HJ (2010) Improving the flavor of fresh fruits: genomics, biochemistry, and biotechnology. New Phytol 187: 44–56. 10.1111/j.1469-8137.2010.03281.x 20456053

[2] Cruz-Hernández A, Paredes-López O (2012) Fruit quality: new insights for biotechnology. Crit Rev Food Sci Nut 52: 272–289. 10.1080/10408398.2010.499844 22214444

[3] McAtee P, Karim S, Schaffer R, David K (2013) A dynamic interplay between phytohormones is required for fruit development, maturation, and ripening. Front Plant Sci 4: 79 10.3389/fpls.2013.00079 23616786 PMC3628358

[4] Yoshimoto K, Jikumaru Y, Kamiya Y, Kusano M, Consonni C, Panstruga R, et al (2009) Autophagy negatively regulates cell death by controlling NPR1-dependent salicylic acid signaling during senescence and the innate immune response in Arabidopsis. Plant Cell 21: 2914–2927. 10.1105/tpc.109.068635 19773385 PMC2768913

[5] Gambetta GA, Matthews MA, Shaghasi TH, McElrone AJ, Castellarin SD (2010) Sugar and abscisic acid signaling orthologs are activated at the onset of ripening in grape. Planta 232: 219–234. 10.1007/s00425-010-1165-2 20407788 PMC2872022

[6] Oikawa A, Otsuka T, Jikumaru Y, Yamaguchi S, Matsuda F, Nakabayashi R, et al (2011) Effects of freeze-drying of samples on metabolite levels in metabolome analyses. J Sep Sci 34: 3561–3567. 10.1002/jssc.201100466 21898815

[7] Oikawa A, Fujita N, Horie R, Saito K, Tawaraya K (2011) Solid-phase extraction for metabolomic analysis of high-salinity samples by capillary electrophoresis-mass spectrometry. J Sep Sci 34: 1063–1068. 10.1002/jssc.201000890 21416606

[8] Lin L-Z, Harnly JM (2008) Phenolic compounds and chromatographic profiles of pear skins (*Pyrus* spp.). J Agr Food Chem 56: 9094–9101. 18778075 10.1021/jf8013487

[9] Murayama H, Katsumata T, Horiuchi O, Fukushima T (2002) Relationship between fruit softening and cell wall polysaccharides in pears after different storage periods. Postharvest Biol Technol 26: 15–21.

[10] Barham D, Trinder P (1972) An improved colour reagent for the determination of blood glucose by the oxidase system. Analyst 97: 142–145. 5037807 10.1039/an9729700142

[11] Oikawa A, Nakamura Y, Ogura T, Kimura A, Suzuki H, Sakurai N, et al (2006) Clarification of pathway-specific inhibition by Fourier transform ion cyclotron resonance/mass spectrometry-based metabolic phenotyping studies. Plant Physiol 142: 398–413. 16905671 10.1104/pp.106.080317 PMC1586045

[12] Caraux G, Pinloche S (2005) PermutMatrix: a graphical environment to arrange gene expression profiles in optimal linear order. Bioinformatics 21: 1280–1281. 15546938 10.1093/bioinformatics/bti141

[13] Toennies G, Homiller RP (1942) The oxidation of amino acids by hydrogen peroxide in formic acid. J Am Chem Soc 64: 3054–3056.

[14] Schram E, Moore S, Bigwood EJ (1954) Chromatographic determination of cystine as cysteic acid. Biochem J 57: 33–37. 13159945 10.1042/bj0570033 PMC1269701

[15] Bradford MM (1976) Rapid and sensitive method for the quantitation of microgram quantities of protein utilizing the principle of protein-dye binding. Anal Biochem 72: 248–254. 942051 10.1016/0003-2697(76)90527-3

[16] Ecker JR (1995) The ethylene signal transduction pathway in plants. Science 268: 667–675. 7732375 10.1126/science.7732375

[17] Østergaard L (2009) Don't 'leaf' now. The making of a fruit. Curr Opin Plant Biol 12: 36–41. 10.1016/j.pbi.2008.09.011 19013099

[18] Mazza G (1995) Anthocyanins in grapes and grape products. Crit Rev Food Sci Nutr 35: 341–371. 7576162 10.1080/10408399509527704

[19] Jacob JK, Tiwari K, Correa-Betanzo J, Misran A, Chandrasekaran R, Paliyath G (2012) Biochemical basis for functional ingredient design from fruits. Ann Rev Food Sci Technol 3: 79–104. 22224553 10.1146/annurev-food-022811-101127

[20] Moco S, Bino RJ, Vorst O, Verhoeven HA, de Groot J, van Beek TA, et al (2006) A liquid chromatography-mass spectrometry-based metabolome database for tomato. Plant Physiol 141: 1205–1218. 16896233 10.1104/pp.106.078428 PMC1533921

[21] Osorio S, Alba R, Nikoloski Z, Kochevenko A, Fernie AR, Giovannoni JJ (2012) Integrative comparative analyses of transcript and metabolite profiles from pepper and tomato ripening and development stages uncovers species-specific patterns of network regulatory behavior. Plant Physiol 159: 1713–1729. 10.1104/pp.112.199711 22685169 PMC3425208

[22] Lombardo VA, Osorio S, Borsani J, Lauxmann MA, Bustamante CA, Budde CO, et al (2011) Metabolic profiling during peach fruit development and ripening reveals the metabolic networks that underpin each developmental stage. Plant Physiol 157: 1696–1710. 10.1104/pp.111.186064 22021422 PMC3327199

[23] Zhang J, Wang X, Yu O, Tang J, Gu X, Wan X, et al (2011) Metabolic profiling of strawberry (*Fragaria x ananassa* Duch.) during fruit development and maturation. J Exp Bot 62: 1103–1118. 10.1093/jxb/erq343 21041374

[24] McDougall G, Martinussen I, Stewart D (2008) Towards fruitful metabolomics: high throughput analyses of polyphenol composition in berries using direct infusion mass spectrometry. J Chrom B 871: 362–369. 10.1016/j.jchromb.2008.06.032 18650134

[25] Zamboni A, Di Carli M, Guzzo F, Stocchero M, Zenoni S, Ferrarini A, et al (2010) Identification of putative stage-specific grapevine berry biomarkers and omics data integration into networks. Plant Physiol 154: 1439–1459. 10.1104/pp.110.160275 20826702 PMC2971619

[26] Son HS, Hwang GS, Kim KM, Ahn HJ, Park WM, Van Den Berg F, et al (2009) Metabolomic studies on geographical grapes and their wines using 1H NMR analysis coupled with multivariate statistics. J Agr Food Chem 57: 1481–1490. 19192969 10.1021/jf803388w

[27] Albers E (2009) Metabolic characteristics and importance of the universal methionine salvage pathway recycling methionine from 5’-methylthioadenosine. IUBMB Life 61: 1132–1142. 10.1002/iub.278 19946895

[28] Ohkama-Ohtsu N, Oikawa A, Zhao P, Xiang C, Saito K, Oliver DJ (2008) A γ-glutamyl transpeptidase-independent pathway of glutathione catabolism to glutamate via 5-oxoproline in Arabidopsis. Plant Physiol 148: 1603–1613. 10.1104/pp.108.125716 18768907 PMC2577253

[29] Takahashi H, Kopriva S, Giordano M, Saito K, Hell R (2011) Sulfur assimilation in photosynthetic organisms: molecular functions and regulations of transporters and assimilatory enzymes. Ann Rev Plant Biol 62: 157–184. 21370978 10.1146/annurev-arplant-042110-103921

[30] Jia HF, Chai YM, Li CL, Lu D, Luo JJ, Qin L, et al (2011) Abscisic acid plays an important role in the regulation of strawberry fruit ripening. Plant Physiol 157: 188–199. 10.1104/pp.111.177311 21734113 PMC3165869

[31] Hoekstra FA, Golovina EA, Buitink J (2001) Mechanisms of plant desiccation tolerance. Trends Plant Sci 6: 431–438. 11544133 10.1016/s1360-1385(01)02052-0

[32] Zhu JK (2002) Salt and drought stress signal transduction in plants. Ann Rev Plant Biol 53: 247–273. 12221975 10.1146/annurev.arplant.53.091401.143329 PMC3128348

[33] Tuteja N (2007) Mechanisms of high salinity tolerance in plants. Methods Enzymol 428: 419–438. 17875432 10.1016/S0076-6879(07)28024-3

[34] Vidya Vardhini B, Rao SS (2002) Acceleration of ripening of tomato pericarp discs by brassinosteroids. Phytochemistry: 61: 843–847. 12453577 10.1016/s0031-9422(02)00223-6

[35] Symons GM, Davies C, Shavrukov Y, Dry IB, Reid JB, Thomas MR (2006) Grapes on steroids. Brassinosteroids are involved in grape berry ripening. Plant Physiol 140: 150–158. 16361521 10.1104/pp.105.070706 PMC1326039

[36] Ashikari M, Matsuoka M (2006) Identification, isolation and pyramiding of quantitative trait loci for rice breeding. Trends Plant Sci 11: 344–350. 16769240 10.1016/j.tplants.2006.05.008

[37] Morreel K, Goeminne G, Storme V, Sterck L, Ralph J, Coppieters W, et al (2006) Genetical metabolomics of flavonoid biosynthesis in Populus: a case study. Plant J 47: 224–237. 16774647 10.1111/j.1365-313X.2006.02786.x

[38] Rowe HC, Hansen BG, Halkier BA, Kliebenstein DJ (2008) Biochemical networks and epistasis shape the Arabidopsis thaliana metabolome. Plant Cell 20: 1199–1216. 10.1105/tpc.108.058131 18515501 PMC2438456

[39] Koulman A, Cao M, Faville M, Lane G, Mace W, Rasmussen S (2009) Semi-quantitative and structural metabolic phenotyping by direct infusion ion trap mass spectrometry and its application in genetical metabolomics. Rapid Comm Mass Spectorom 23: 2253–2263. 10.1002/rcm.4142 PMC2970905 19551846

[40] Matsuda F, Okazaki Y, Oikawa A, Kusano M, Nakabayashi R, Kikuchi J, et al (2012) Dissection of genotype-phenotype associations in rice grains using metabolome quantitative trait loci analysis. Plant J 70: 624–636. 10.1111/j.1365-313X.2012.04903.x 22229385

[41] Wu J, Wang Z, Shi Z, Zhang S, Ming R, Zhu S, et al (2013) The genome of the pear (*Pyrus bretschneideri* Rehd.). Genome Res 23: 396–408. 10.1101/gr.144311.112 23149293 PMC3561880

