# Supplementary figures and images for: Metabolic Profiling of Developing Pear Fruits Reveals Dynamic Variation in Primary and Secondary Metabolites, Including Plant Hormones
Source: PLoS One. 2015 Jul 13;10(7):e0131408. doi: 10.1371/journal.pone.0131408 (PMC4500446; doi:10.1371/journal.pone.0131408)

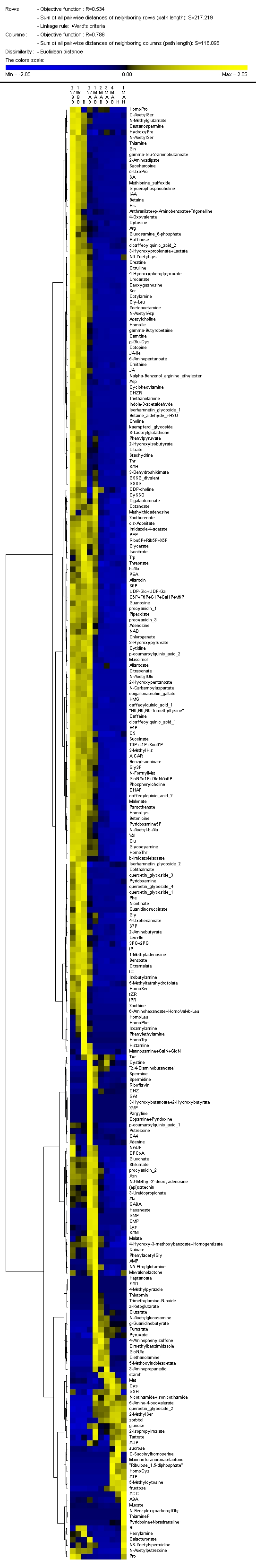

Supplement: S1 Fig — Yellow and blue colors represent higher and lower amounts of metabolites, respectively. (JPG) [file pone.0131408.s001.jpg]
